# High expression of GNA13 is associated with poor prognosis in hepatocellular carcinoma

**DOI:** 10.1038/srep35948

**Published:** 2016-11-24

**Authors:** Yi Xu, Jian Rong, Shiyu Duan, Cui Chen, Yin Li, Baogang Peng, Bin Yi, Zhousan Zheng, Ying Gao, Kebing Wang, Miao Yun, Huiwen Weng, Jiaxing Zhang, Sheng Ye

**Affiliations:** 1 Department of Oncology, The First Affiliated Hospital, Sun Yat-Sen University, Guangzhou 510080, China; 2 Department of Extracorporeal Circulation, The First Affiliated Hospital, Sun Yat-sen University, Guangzhou 510080, China; 3 Department of Pathology, Nanfang Hospital, Southern Medical University, Guangzhou 510515, China; 4 Department of Pathology, School of Basic Medical Sciences, Southern Medical University, Guangzhou 510515, China; 5 Department of Gastrointestinal Surgery, The First Affiliated Hospital, Sun Yat-sen University, Guangzhou 510080, China; 6 Department of Liver Surgery, The First Affiliated Hospital, Sun Yat-sen University, Guangzhou 510080, China; 7 Department of Surgical Laboratory, The First Affiliated Hospital, Sun Yat-Sen University, Guangzhou 510080, China; 8 Sun Yat-sen University Cancer Center, State Key Laboratory of Oncology in South China, Collaborative Innovation Center for Cancer Medicine, Guangzhou 510060, China; 9 Department of Ultrasound, Cancer Center, Sun Yat-Sen University, Guangzhou 510060, China

## Abstract

Guanine nucleotide binding protein alpha 13 (GNA13) has been found to play critical roles in the development of several human cancers. However, little is known about GNA13 expression and its clinical significance in hepatocellular carcinoma (HCC). In our study, GNA13 was reported to be significantly up-regulated in HCC tissues, and this was correlated with several clinicopathological parameters, including tumor multiplicity (P = 0.004), TNM stage (P = 0.002), and BCLC stage (P = 0.010). Further Cox regression analysis suggested that GNA13 expression was an independent prognostic factor for overall survival (P = 0.014) and disease-free survival (P = 0.005). Moreover, we found that overexpression of GNA13 couldn’t promote cell proliferation *in vitro*, but could significantly increase the invasion ability of HCC cells. Together, our study demonstrates GNA13 may be served as a prognostic biomarker for HCC patients after curative hepatectomy, in which high expression of GNA13 suggests poor prognosis of HCC patients.

Hepatocellular carcinoma (HCC), one of the most common cancer in the world, is the second leading cause of cancer-related death worldwide in men^1^. Despite short-term improvements made by curative hepatectomy along with other surgical and therapeutic approaches, such as local ablation therapy and transarterial chemoembolization, the overall survival remains unsatisfactory in HCC patients due to high recurrence and metastasis rates after curative hepatectomy^2^,^3^.

Over recent decades, various molecular biomarkers for early detection and additional prognostic information in HCC have been explored and investigated. For instance, serum a-fetoprotein (AFP) is regarded as the most widely accepted serum marker for its diagnostic and predictive value in HCC patients. In addition, new biomarkers in HCC, such as des-*y*-carboxyprothrombin (DCP), glypican3, osteopontin, and Golgi protein 73 (GP73), have also been found and shown to be of some clinical value^4^,^5^,^6^,^7^. However, the search for specific biomarkers that can evaluate the recurrence and prognosis of HCC and guide molecular targeting therapy in HCC remains substantially limited. Therefore, it is imperative to pay more attention to the investigation of novel specific markers for predicting tumorigenesis, metastasis, recurrence and prognosis for HCC patients.

G protein-coupled receptors(GPCRs) are one of the most significant classes of cell surface receptors and play critical roles in cell physiology^8^,^9^. Heterotrimeric G proteins, consisting of Gα-subunit, Gβ-subunit and Gγ-subunit, can mediate signaling through specific GPCRs. Among all Gα families (namely Gs, Gi, Gq and G12), the G12 subfamily, consisting of Gα12(GNA12) and Gα13(GNA13), has been of particular interest to oncologists due to their roles in promoting oncogenic transformation and tumor cell growth^10^,^11^,^12^. Previous studies have found that GNA12 and GNA13 proteins are up-regulated in several human cancers, and the GNA12/GNA13 signaling may exhibit a crucial role in cancer cell invasion and metastasis^13^,^14^,^15^. Of note, there are more studies which focus on the role of GNA12 in cancer biology, while less was studied about the specific role of GNA13. Recently, increased expression of GNA13 has been found in several types of malignancies, especially in more aggressive breast and prostate cancer. Furthermore, the impact of this upregulation could contribute to cancer cell invasion and migration^16^,^17^. Li and the colleagues found GNA13 played a critical role in lysophosphatidic acid (LPA)-stimulated invasive migration of pancreatic cancer cells^18^. We previously observed that upregulation of GNA13 could promote the tumorigenicity and proliferation of gastric cancer(GC) cells^19^. In addition, elevated expression of GNA13 was reported to exert proliferative effects in human small cell lung cancer cells^20^. However, to date, the expression status of GNA13 in HCC and its significance remains largely elusive.

In this study, qRT-PCR, western blotting, and immunohistochemistry (IHC) were performed to examine the mRNA and protein levels of GNA13 in HCC, and the relationship between GNA13 expression and various clinicopathologic parameters was evaluated in order to systematically investigate the clinicopathological and prognostic impacts of GNA13 in HCC patients.

## Results

### Detection of GNA13 expression in HCC based on western blotting and qPCR

**Figure 1:**
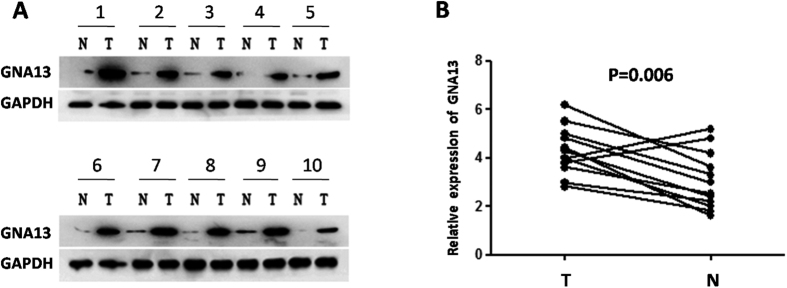
The expression of GNA13 in HCC by Western blotting and qPCR. (**A**) Among 12 HCC cases, increased expression of GNA13 was detected via western blotting in 10 pairs of HCC tissues compared with the matched non-cancerous liver tissues. The expression levels were normalised to those of GAPDH. (**B**) The mRNA expression of GNA13 was significantly upregulated in 10/12 pairs of HCC tissues based on qPCR. The expression levels were normalised to those of GAPDH. N, adjacent normal liver tissue; T, HCC tissue.

Western blotting was employed to examine the expression of GNA13 in 12 pairs of matched HCC samples and adjacent non-cancerous tissues. As described in Fig. 1A, among the total fresh samples, 10 HCC samples exhibited higher GNA13 expression compared to that in the corresponding normal tissues. We also used qPCR to detect the GNA13 mRNA expression level in the 12 pairs of tissues. The qPCR revealed consistent results, where mRNA expression of GNA13 in HCC tissue was obviously higher than that in adjacent non-cancerous liver tissues (P = 0.006) (Fig. 1B).

### Detection of GNA13 expression in HCC by immunohistochemistry(IHC)

**Figure 2:**
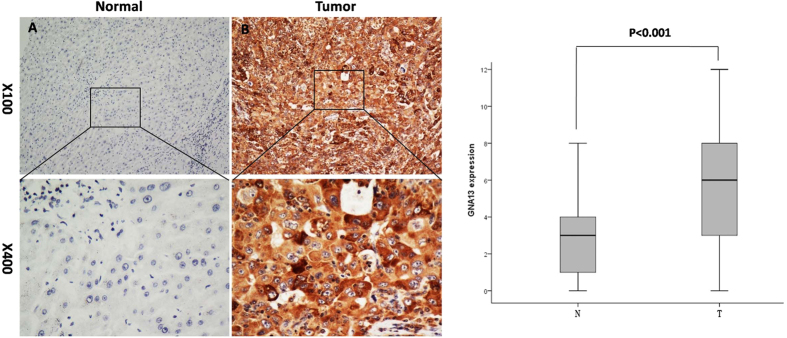
Representative images of GNA13 expression in adjacent non-cancerous liver tissues and HCC tissues via IHC. GNA13 was absent or only weakly detected in adjacent normal liver cells (**A**), whereas its upregulation was mainly detected in HCC tissues (**B**) (original magnification, x100 and x400)(Left). The box plot showed the mean staining score of GNA13 in HCC tissues (T) and the adjacent non-cancerous liver tissues (N) (P < 0.001)(Right).

**Figure 3:**
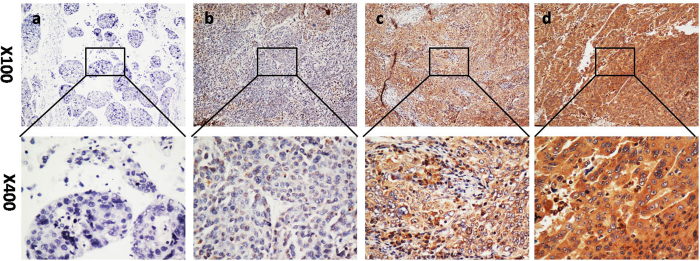
The protein expression of GNA13 in HCC by immunohistochemistry. The representative images show different staining intensities of GNA13: (**a**) negative staining, (**b**) weak staining, (**c**) moderate staining, and (**d**) strong staining (original magnification, x100 and x400).

The GNA13 expression level in 246 pairs of HCC and adjacent non-tumorous liver tissues was further examined by IHC (Fig. 2). As shown in Fig. 2B, high expression of GNA13 protein was detected in 148/246(60.2%) of HCC tissues. Furthermore, high levels of GNA13 were mainly found in the cytoplasm of carcinoma cells. In contrast, weak or negative GNA13 staining was observed in adjacent normal liver tissues (Fig. 2A). The four categories of the intensity of GNA13 immunostaining are described in Fig. 3. According to immunohistochemical scores(IHC scores), the patients were divided into two groups: low GNA13 expression group (IHC score ≤ 4) and high GNA13 expression group (IHC score > 4).

### The association between GNA13 expression and clinicopathological parameters

Pearson’s chi-square (χ2) test/Fisher’s exact test was performed to investigate the association between GNA13 expression and clinicopathological characteristics in 246 cases with HCC. Our analyses showed significant correlations between GNA13 expression and three characteristics including tumor multiplicity (P = 0.004), TNM stage (P = 0.002) and BCLC stage (P = 0.010). However, we found no statistically significant correlations between GNA13 expression and the rest of clinicopathological features, such as patient age, gender, AFP, HBsAg, Liver cirrhosis, tumor size, pathological grades, and vascular invasion (P > 0.05, Table 1).

### The relationship of high GNA13 expression with poor survival in HCC patient

**Figure 4:**
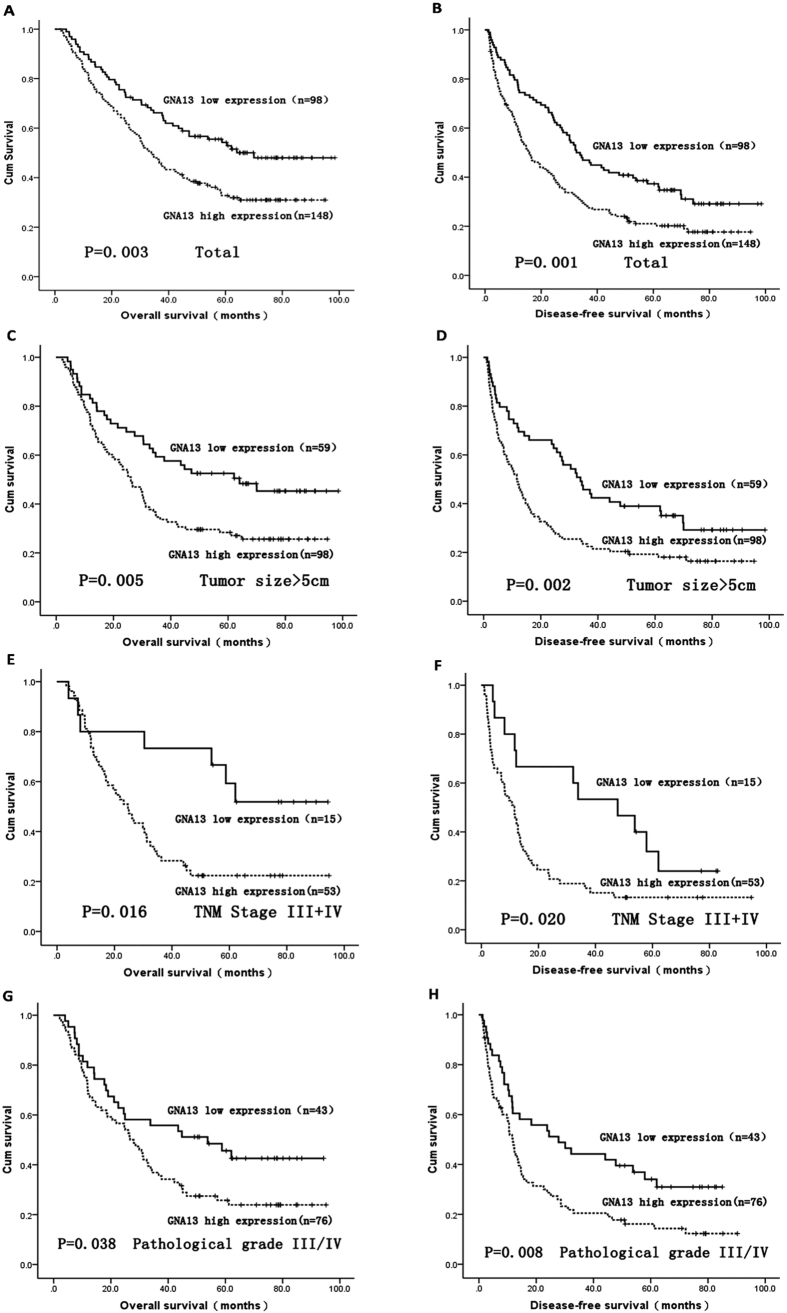
Survival analysis of GNA13 expression by Kaplan-Meier method. Overall survival rate and disease-free survival rate in total (**A**,**B**) HCC patients with low/high GNA13 expression. Overall survival rate and disease-free survival rate in tumor size > 5 cm (**C**,**D**), TNM stage III/IV (**E**,**F**), and pathological grade III/IV (**G**,**H**) HCC patients with low/high GNA13 expression.

The relationship between GNA13 expression in HCC patients and the survival time of these patients was analyzed by Kaplan-Meier analysis and the log-rank test (Fig. 4). The log-rank test showed that the survival time was different between high and low GNA13 expression groups. The median overall survival (OS) time of high GNA13 expression group was 39.1 months, remarkably shorter than that of low GNA13 expression group (48.9 months) (log-rank test, P = 0.003, Fig. 4A). Furthermore, the patients with high GNA13 expression exhibited a significantly shorter DFS than those exhibiting low expression of GNA13 (log-rank test, P = 0.001, Fig. 4B). Moreover, we deployed survival analysis towards the level of GNA13 expression in subgroups of HCC patients against tumor size, TNM stages, and histological grades. Our results revealed that high expression of GNA13 could be a prognostic factor for HCC patients with tumor size >5 cm (OS: P = 0.005, Fig. 4C; DFS: P = 0.002, Fig. 4D), TNM stage III/IV (OS: P = 0.016, Fig. 4E; DFS: P = 0.020, Fig. 4F), and pathological grade III/IV (OS: P = 0.038, Fig. 4G; DFS:P = 0.008, Fig. 4H).

In addition, the prognostic value of GNA13 expression for OS and DFS was analyzed using a univariate analysis model on these clinialpathological parameters (Tables 2 and 3). Based on this analysis, GNA13 expression, tumor size, tumor multiplicity, liver cirrhosis, pathological grades, TNM stage, BCLC stage and vascular invasion were found to impact survival. Next, these statistically significant parameters were further examined using a multivariate Cox regression analysis to evaluate the significance of GNA13 expression in HCC prognosis (Tables 2 and 3). We observed that GNA13 expression, pathological grades and tumor size were independent prognostic factors for OS, whereas GNA13 expression, as well as pathological grades and liver cirrhosis, were independent prognostic factors for DFS. Therefore, the results suggested that the GNA13 expression level was significantly associated with the prognosis of HCC.

### Effect of GNA13 overexpression on cell proliferation and invasion

**Figure 5:**
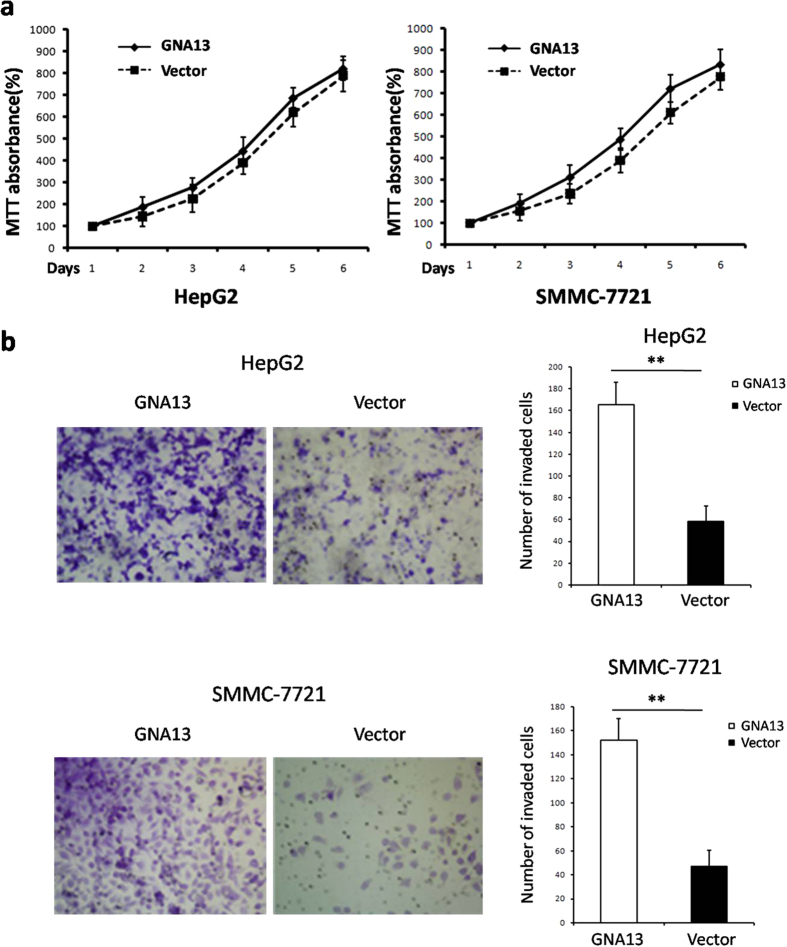
Effect of GNA13 overexpression on cell proliferation and invasion *in vitro*. **(a)** Overexpression of GNA13 could not promote the proliferation of HepG2 and SMMC-7721 cells *in vitro* as determined by MTT assay. **(b)** In the invasion assay, the cell invasion ability was significantly increased after overexpression of GNA13 in HepG2 and SMMC-7721 cells when compared with Vector control. Bars represent the mean ± SD of three independent experiments. *P < 0.05.

To determine the effect of GNA13 on cell proliferation and invasion in HCC cells, we established stable GNA13-overexpressing HCC cells, HepG2 and SMMC-7721. In the MTT assay, overexpression of GNA13 could not promote cell proliferation in both HepG2 and SMMC-7721 cells *in vitro* compared to the vector controls group (Fig. 5a). By contrast, the invasion assay proved that the cell invasion ability was significantly increased after overexpression of GNA13 expression in HepG2 and SMMC-7721 cells when compared with vector controls (Fig. 5b).

### Effect of GNA13 overexpression on the expression of P-AKT and P-ERK

To investigate the possible oncogenic pathways that may involve the function of GNA13, western blotting was performed. The results showed that overexpression of GNA13 had no obvious effect on the protein levels of P-AKT and P-ERK in HepG2 and SMMC-7721 cells (Supplementary Figure S1). Our data suggest that GNA13 overexpression in HCC cells led to cancer progression and tumorigenesis via other signalling pathways.

## Discussion

To our knowledge, clinical/pathological staging is the most commonly and widely used predictive methods for the prognosis of patients with HCC. However, the prognosis of HCC patients with the same clinical/pathological stage often deviates after curative hepatectomy, and this discrepancy is usually unexplained. Thus, it is useful to find new biomarkers for the prognosis and optimal treatment strategies of HCC.

Recently, many GPCRs and their respective ligands have been shown to correlate with tumor formation and organ-specific metastasis in several types of malignancies^21^. These GPCRs can signal through heterotrimeric G proteins, especially the GNA12/GNA13 subfamily which has been shown to mediate cancer cell invasion and metastasis^22^,^23^,^24^,^25^,^26^. It was reported that GAN13 had been closely associated with tumor progression in different types of human cancers, such as prostate, breast, colorectal, pancreatic and gastric cancers^16^,^17^,^19^,^20^,^27^. These findings reveal a potential carcinogenic role of GNA13 in multiple human malignancies. To date, however, the expression status of GNA13 in HCC and its relationship with the clinicopathological parameters have not been elucidated. In a small test at the beginning of the present study, western blotting and qPCR were performed to examine the expression level of GNA13 in several paired HCC and adjacent non-neoplastic tissues. GNA13 expression was apparently upregulated at the protein and mRNA level in 10 out of 12 cases. The expression of GNA13 protein was then evaluated by immunohistochemistry(IHC) in an expanded population with 246 pairs of HCC samples. We observed that the GNA13 protein was predominantly detected in the cytoplasm of HCC and was increased in 60.2% of paraffin-embedded HCC tissues. By contrast, the normal liver tissues presented mainly negative expression of GNA13. These findings suggest that upregulation of GNA13 expression may provide a selective advantage in the HCC tumorigenic processes.

Previous data suggested that GNA13 was significantly upregulated in more aggressive breast cancer cells, and elevated GNA13 expression might be used as a potential marker for breast cancer progression^16^. We reported that GNA13 was upregulated in gastric cancer(GC), and GNA13 upregulation was closely associated with aggressive characteristic of cancer progression and poor survival in GC patients^19^. In the current study, we provided evidence that increased expression of GNA13 was significantly associated with invasive characteristics of HCC, including *multiple liver lesions*, advanced TNM stage, and BCLC stage. Importantly, the Kaplan-Meier curve and multivariate Cox regression analysis were performed to show that high GNA13 expression was identified as an independent predictor for shorter OS and DFS in HCC. Based on the subgroup analysis, GNA13 predicted the clinical outcome of the following subsets of HCC patients: tumor size > 5 cm, TNM stage III/IV, and pathological grade III/IV. Therefore, high GNA13 expression seems to have the potential to predict poor OS and DFS outcomes in patients with HCC. The detection of GNA13 expression status might be served as an integrated approach for identifying HCC patients at high risk of cancer progression. Thus, HCC patients with high GNA13 expression should be paid much more attention to and/or should be more closely followed up after surgical resection.

GNA13 has been demonstrated to be an important regulator of cancer cell proliferation, invasion, migration and metastasis^18^,^28^,^29^,^30^. It has been reported that GNA13 expression is regulated via post-transcriptional mechanisms involving some microRNAs in prostate and breast cancer cells^16^,^17^. The deregulation of microRNAs has been associated with tumorigenesis, invasion, and metastasis in several human tumors^31^,^32^. Moreover, elevated expression of GNA13 promoted colorectal cancer metastasis by triggering the epithelial-mesenchymal transition (EMT)^27^. A number of previous studies indicate that GNA12/13 proteins have the potential to mediate cancer cell invasion and metastasis via activating RhoA^14^,^33^,^34^. Previously, we also underlined that increased expression of GNA13 could accelerate the G1/S phase transition, thereby promoting cancer cell proliferation and tumorigenesis probably due to modulation of c-Myc and FOXO1 activity^19^. However, loss of GNA13 triggered the growth and dissemination of germinal center B cells, and thus contributing to the development and progression of germinal center B cell-derived lymphoma^35^. These seemingly contradictory results suggested that abnormal expression of GNA13 in different types of tumors might affect different molecular signal pathways. In our study, overexpression of GNA13 couldn’t promote cell proliferation *in vitro*, but could significantly promote cell invasion in HCC cell lines. Additionally, we found that overexpression of GNA13 had no obvious effect on the protein levels of P-AKT and P-ERK (Supplementary Figure S1), suggesting that GNA13 might affect HCC progression via other pathways. All these data implied that the role of GNA13 in human cancer is tissue-specific. Although we observed that high expression of GNA13 in HCC was closely implicated in tumor progression, further investigation is needed to fully elucidate the precise mechanisms of GNA13 involved in the metastasis and progression of HCC.

In summary, our study demonstrated for the first time that GNA13 was highly expressed in HCC, and increased GNA13 expression was closely associated with unfavorable prognosis in HCC. These data indicated the expression levels of GNA13, as detected by IHC, could be a potential biomarker for poor differentiation and a useful predictor for unfavourable prognosis of HCC patients after curative hepatectomy.

## Materials and Methods

### Patients and tissue specimens

For analysis of mRNA and protein expression, fresh tumor tissue samples and matched adjacent non-cancerous liver tissue samples from 12 HCC patients were obtained in operation from the First Affiliated Hospital, Sun Yat-Sen University, Guangzhou, China. These resected tissue samples were rapidly frozen and stored at a −80 °C until they were used for qPCR and western blotting. Paraffin-embedded specimens of 246 HCC patients from Sun Yat-Sen University Cancer Center between February 1999 and June 2002, as well as clinical and pathological data (such as gender, age, tumor size, AFP, HBsAg, liver cirrhosis, tumor multiplicity, pathological stage, TNM stage, BCLC stage and vascular invasion), were obtained to expand the study. None of these patients had distant metastasis or received any anti-tumor treatments before operation. The follow-up data were obtained by telephone or from the outpatient records. Patients with unknown cause of death were excluded. Among the 246 HCC patients aged from 17 to 75 years (mean, 49 years), there were 225 males (91.5%) and 21 females (8.5%). The clinicopathological parameters for these patients were summarized in Table 1. The tumor stages were defined according to the seventh edition of the American Joint Committee on Cancer Staging manual and the 2011 edition of the Barcelona Clinic Liver Cancer (BCLC) staging system. The pathological grade of tumor differentiation was evaluated according to the criteria proposed by Edmonson and Steiner. The study was approved by the Ethics Committee of Sun Yat-sen University and written informed consent was obtained from all patients. All experimental methods were carried out in accordance with approved guidelines of Sun Yat-Sen University.

### Quantitative real-time polymerase chain reaction (qPCR)

Total RNA was extracted from 12 pairs of HCC tissues and matched adjacent non-malignant liver tissues using Trizol regent (Invitrogen, Grand Island, NY, USA) and cDNA was synthesized by SuperScript *Reverse Transcriptase* kit (Promega, Madison, WI, USA) according to the manufacturer’s instruction. The primer sequences used to amplify GNA13 were: TCGGGAAAAGACCTATGTGAA (forward) and CAACCAGCACCCTCATACCT (reverse). GAPDH was used as an internal control for normalization.

### Western blotting

12 pairs of fresh HCC tissues, the matched adjacent non-tumorous liver tissues and HCC cells (HepG2 and SMMC-7721) were lysed in a RIPA lysis buffer, and lysates were cleared by centrifugation (12,000 rpm, 30 min, 4 °C), respectively. Afterwards, the supernatant was collected and mixtures of proteins was separated by SDS-polyacrylamide gel electrophoresis (PAGE), and subsequently transferred onto a polyvinylidene difluoride (PVDF) membrane (Pall Corp., Port Washington, NY). The membrane was blocked with 5% skimmed milk for 1 h and then incubated with primary mouse monoclonal antibodies against GNA13, P-AKT, P-ERK, and GAPDH (1:1000 dilution; Abcam, Cambridge, MA, USA) at 4 °C overnight. After thoroughly washing the membrane, it was then incubated with the secondary anti-mouse antibody from Santa Cruz Biotechnology (Santa Cruz Biotechnology, CA, USA) for 1 h at room temperature. Finally, the immunoreactive signals were detected by means of an enhanced chemiluminescence (ECL) Western blotting detection protocol.

### Immunohistochemistry

Immunohistochemical analysis was carried out to examine the GNA13 expression levels with a standard two-step method in 246 HCC tissue specimens. The paraffin-embedded HCC specimens were cut into 5-μm sections and placed in an oven at 65˚C for 2 h. The sections were then deparaffinised in xylene and hydrated through a series of graded ethanol. Subsequently, the sections were immersed in 3% hydrogen peroxide for 10 min to block the endogenous peroxidase activity. To retrieve the antigenicity, the slides were then boiled in citrate buffer solution (pH 6.5) for 20 min in a micro-wave oven. After washing three times for 5 min in phosphate buffered saline (PBS), the sections were incubated with a primary antibody against GNA13 (1:500 dilution; Abcam, Cambridge, MA, USA) at 4˚C overnight. After rinsing three times for 5 min in PBS, the tissue sections were sequentially incubated with a secondary antibody for 1 hour at room temperature. After three further washes in PBS, the sections were stained with 3,3-diaminobenzidine (DAB), counterstained with Mayer’s haematoxylin, dehydrated and mounted. Slides with positive immunohistochemical staining were considered as the positive controls. The slides were immunoreacted with PBS for use as negative controls.

### Immunohistochemical (IHC) evaluation

The evaluation of IHC results was performed by three independent pathologists. Cytoplasmic immunoreactivity for the GNA13 protein was scored by evaluating the intensity of staining and the proportion of positive tumor cells. In brief, the intensity of staining was graded as follows: 0 (no staining), 1 (weakly stained, light yellow), 2 (moderately stained, yellow brown), and 3 (strongly stained, brown). We scored the proportion of positively stained tumor cells according to the following standard: 0 (0%), 1 (1–10%), 2 (11–50%), 3 (51–80%), and 4 (81–100%). Then, the IHC scores were obtained by multiplying the intensity and the proportion scores. Based on this method, the IHC scores were finally classified as follow: “−” (IHC scores 0), “+” (IHC scores 1–4), “++” (IHC scores 5–8), and “+++” (IHC scores 9–12). For the purpose of statistical analysis, the cohort of 246 patients with HCC was divided into low GNA13 expression group (“−”, “+”) and high GNA13 expression group (“++”, “+++”).

### Cell proliferation assay

The vector construction was detailed previously^19^. Cell proliferation was analyzed using a 3-(4, 5-dimethylthiazol-2-yl)-175 2, 5-diphenyl tetrazolium bromide (MTT) assay (Sigma). Briefly, cells were seeded in 96-well plates and cultured. Cell proliferation was examined by following standard procedures. Experiments were performed in triplicate.

### Transwell invasion assay

About harvested cells (1 × 10^5^) in 100 μl of serum-free DMEM were added into the upper compartment of 8-μm-pore Transwells (Costar, Corning, Cambridge, MA, USA) and 200 μl of 10% FBS in free medium (Gibco, Invitrogen, Carlsbad, CA, USA) was placed in the lower compartment. The cells were allowed to migrate for 24 h of incubation at 37 °C. For quantification, the cells in the lower compartment were stained with haematoxylin and counted in five randomly selected visual fields (x200 magnification) under a microscope. The experiment was repeated 3 times.

### Statistical analysis

Statistical analyses were performed with SPSS v19.0 (IBM). P-values less than 0.05 were considered to be statistically significant. Pearson’s chi-square (χ2) test/Fisher’s exact test was utilized to evaluate the association between GNA13 expression and the clinicopathological characteristics. OS (Overall Survival) was defined as the interval between date of surgery and date of death or between date of surgery and date of the last observation. DFS (Disease-Free Survival) was defined as the time from date of surgery to date of cancer progression, metastasis, or death. Estimates of the cumulative survival distributions were calculated by the Kaplan-Meier method, and the differences between the two groups were compared using the log-rank test. The significance of various clinicopathological variables was evaluated using univariate and multivariate Cox proportional hazard regression model. The variables that were considered to be significant based on the univariate regression model were selected and analyzed by the multivariate Cox regression model.

## Additional Information

**How to cite this article**: Xu, Y. *et al*. High expression of GNA13 is associated with poor prognosis in hepatocellular carcinoma. *Sci. Rep*. **6**, 35948; doi: 10.1038/srep35948 (2016).

**Publisher’s note:** Springer Nature remains neutral with regard to jurisdictional claims in published maps and institutional affiliations.

## Supplementary Material

Supplementary Information

## Figures and Tables

**Table 1:** Correlation between GNA13 expression and clinical and pathological characters in HCC patients.

Variable	All cases N = 246 (%)	Low GNA13 expression N = 98 (%)	High GNA13 expression N = 148 (%)	χ^2^	P value
Age (years)^a^				0.329	0.566
≤49	121 (49.2)	46 (38.0)	75 (62.0)		
>49	125 (50.8)	52 (41.6)	73 (58.4)		
Mean ± SD (49.4 ± 12.1)					
Gender				1.216	0.270
Female	21 (8.5)	6 (28.6)	15 (71.4)		
Male	225 (91.5)	92 (40.9)	133 (59.1)		
HBsAg				0.665	0.415
Yes	216 (87.8)	84 (38.9)	132 (61.1)		
No	30 (12.2)	14 (46.7)	16 (53.3)		
AFP (ng/ml)				1.981	0.159
<200	112 (45.5)	50 (44.6)	62 (55.4)		
≥200	134 (54.5)	48 (35.8)	86 (64.2)		
Liver cirrhosis				3.413	0.065
Yes	191 (77.6)	82 (42.9)	109 (57.1)		
No	55 (22.4)	16 (29.1)	39 (70.9)		
Tumor size (cm)				0.923	0.337
≤5	89 (36.2)	39 (43.8)	50 (56.2)		
>5	157 (63.8)	59 (37.6)	98 (62.4)		
Tumor multiplicity				8.400	0.004*
Single	202 (82.1)	89 (44.1)	113 (55.9)		
Multiple	44 (17.9)	9 (20.5)	35 (79.5)		
Pathological grade				2.705	0.439
Well (I)	20 (8.1)	11 (55.0)	9 (45.0)		
Moderate (II)	107 (43.5)	44 (41.1)	63 (58.9)		
Poor (III)	114 (46.4)	41 (36.0)	73 (64.0)		
Undifferentiated (IV)	5 (2.0)	2 (40.0)	3 (60.0)		
TNM stage				14.644	0.002*
I	160 (65.0)	77 (48.1)	83 (51.9)		
II	18 (7.3)	6 (33.3)	12 (66.7)		
III	56 (22.8)	11 (19.6)	45 (80.4)		
IV	12 (4.9)	4 (33.3)	8 (66.7)		
BCLC stage				11.295	0.010*
0	9 (3.7)	6 (66.7)	3 (33.3)		
A	171 (69.5)	76 (44.4)	95 (55.6)		
B	38 (15.4)	8 (21.1)	30 (78.9)		
C	28 (11.4)	8 (28.6)	20 (71.4)		
Vascular invasion				2.514	0.113
Yes	18 (7.3)	4 (22.2)	14 (77.8)		
No	228 (92.7)	94 (41.2)	134 (58.8)		

^a^patients were divided according to the median age; AFP: alpha-fetoprotein; HBsAg: hepatitis B surface antigen.

^*^P < 0.05.

**Table 2:** Univariate and multivariate Cox regression analysis of prognostic factors in 246 HCC patients for overall survival.

Variable	Univariate analysis			Multivariate analysis		
	HR	95% CI	P-value	HR	95% CI	P-value
**Overall survival**						
Age (years)						
>49 vs. ≤49	1.005	0.991–1.019	0.504			
Sex						
male vs. female	0.670	0.353–1.274	0.222			
AFP (ng/ml)						
≥200 vs. <200	1.214	0.878–1.679	0.241			
Liver cirrhosis						
Positive vs. Negative	1.226	0.821–1.830	0.320			
Tumor size(cm)						
>5 vs. ≤5	1.704	1.200–2.421	0.003*	1.596	1.110–2.294	0.012*
Tumor multiplicity						
Multiple vs. Single	1.787	1.218–2.622	0.003*	1.864	0.831–4.183	0.131
Pathological grade						
III-IV vs. I-II	1.685	1.218–2.331	0.002*	1.604	1.145–2.248	0.006*
TNM stage						
III-IV vs. I-II	1.583	1.121–2.235	0.009*	0.961	0.580–1.593	0.879
BCLC stage						
B-C vs. 0-A	1.736	1.229–2.452	0.002*	0.774	0.324–1.852	0.565
Vascular invasion						
Positive vs. Negative	1.887	1.065–3.343	0.029*	2.035	0.849–4.881	0.111
GNA13 expression						
High vs. Low	1.676	1.187–2.366	0.003*	1.558	1.096–2.215	0.014*

^*^P < 0.05.

**Table 3:** Univariate and multivariate Cox regression analysis of prognostic factors in 246 HCC patients for disease-free survival.

Variable	Univariate analysis			Multivariate analysis		
	HR	95% CI	P-value	HR	95% CI	P-value
**Disease-free survival**						
Age (years)						
>49 vs. ≤49	1.002	0.989–1.015	0.782			
Sex						
male vs. female	0.669	0.372–1.202	0.178			
AFP (ng/ml)						
≥200 vs. <200	1.200	0.898–1.604	0.217			
Liver cirrhosis						
Positive vs. Negative	1.527	1.051–2.219	0.026*	1.747	1.190–2.564	0.004*
Tumor size(cm)						
>5 cm vs. ≤5 cm	1.411	1.040–1.915	0.027*	1.379	1.000–1.901	0.050
Tumor multiplicity						
Multiple vs. Single	1.956	1.375–2.782	<0.001*	1.495	0.619–3.612	0.371
Pathological grade						
III-IV vs. I-II	1.489	1.114–1.991	0.007*	1.457	1.079–1.969	0.014*
TNM stage						
III-IV vs. I-II	1.633	1.193–2.234	0.002*	0.972	0.630–1.502	0.900
BCLC stage						
B-C vs. 0-A	2.011	1.471–2.748	<0.001*	1.190	0.475–2.979	0.710
Vascular invasion						
Positive vs. Negative	2.229	1.330–3.737	0.002*	1.714	0.638–4.605	0.285
GNA13 expression						
High vs. Low	1.654	1.222–2.238	0.001*	1.564	1.148–2.132	0.005*

^*^P < 0.05.

## References

[b1] Torre L. A. . Global cancer statistics, 2012. CA Cancer J Clin 65, 87 (2015). 25651787 10.3322/caac.21262

[b2] Yang L. Y. . Mesohepatectomy for centrally located large hepatocellular carcinoma: Indications, techniques, and outcomes. Surgery 156, 1177 (2014). 25444316 10.1016/j.surg.2014.05.012

[b3] Liao W. . High KIF18A expression correlates with unfavorable prognosis in primary hepatocellular carcinoma. Oncotarget 5, 10271 (2014). 25431949 10.18632/oncotarget.2082 PMC4279371

[b4] Shang S. . Identification of osteopontin as a novel marker for early hepatocellular carcinoma. Hepatology 55, 483 (2012). 21953299 10.1002/hep.24703 PMC3914762

[b5] Nakamura S. . Sensitivity and specificity of des-gamma-carboxy prothrombin for diagnosis of patients with hepatocellular carcinomas varies according to tumor size. Am J Gastroenterol 101, 2038 (2006). 16848811 10.1111/j.1572-0241.2006.00681.x

[b6] Marrero J. A. . GP73, a resident Golgi glycoprotein, is a novel serum marker for hepatocellular carcinoma. J Hepatol 43, 1007 (2005). 16137783 10.1016/j.jhep.2005.05.028

[b7] Zhang B. & Yang B. Combined alpha fetoprotein testing and ultrasonography as a screening test for primary liver cancer. J Med Screen 6, 108 (1999). 10444731 10.1136/jms.6.2.108

[b8] Dorsam R. T. & Gutkind J. S. G-protein-coupled receptors and cancer. Nat Rev Cancer 7, 79 (2007). 17251915 10.1038/nrc2069

[b9] Wettschureck N. & Offermanns S. Mammalian G proteins and their cell type specific functions. Physiol Rev 85, 1159 (2005). 16183910 10.1152/physrev.00003.2005

[b10] Yang Y. M. . G 12/13 inhibition enhances the anticancer effect of bortezomib through PSMB5 downregulation. Carcinogenesis 31, 1230 (2010). 20478922 10.1093/carcin/bgq097

[b11] Chan A. M. . Expression cDNA cloning of a transforming gene encoding the wild-type G alpha 12 gene product. Mol Cell Biol 13, 762 (1993). 8423800 10.1128/mcb.13.2.762-768.1993 PMC358958

[b12] Xu N., Bradley L., Ambdukar I. & Gutkind J. S. A mutant alpha subunit of G12 potentiates the eicosanoid pathway and is highly oncogenic in NIH 3T3 cells. Proc Natl Acad Sci USA 90, 6741 (1993). 8393576 10.1073/pnas.90.14.6741 PMC47008

[b13] Cheong S. C. . Gene expression in human oral squamous cell carcinoma is influenced by risk factor exposure. Oral Oncol 45, 712 (2009). 19147396 10.1016/j.oraloncology.2008.11.002

[b14] Kelly P. A Role for the G12 Family of Heterotrimeric G Proteins in Prostate Cancer Invasion. J Biol Chem 281, 26483 (2006). 16787920 10.1074/jbc.M604376200

[b15] Kelly P. . The G12 family of heterotrimeric G proteins promotes breast cancer invasion and metastasis. Proc Natl Acad Sci USA 103, 8173 (2006). 16705036 10.1073/pnas.0510254103 PMC1472448

[b16] Rasheed S. A. K. . MicroRNA-31 controls G protein alpha-13 (GNA13) expression and cell invasion in breast cancer cells. Molecular Cancer 14 (2015). 10.1186/s12943-015-0337-x PMC4379695 25889182

[b17] Rasheed S. A. K., Teo C. R., Beillard E. J., Voorhoeve P. M. & Casey P. J. MicroRNA-182 and MicroRNA-200a Control G-protein Subunit -13 (GNA13) Expression and Cell Invasion Synergistically in Prostate Cancer Cells. J Biol Chem 288, 7986 (2013). 23329838 10.1074/jbc.M112.437749 PMC3597835

[b18] Gardner J. A., Ha J. H., Jayaraman M. & Dhanasekaran D. N. The gep proto-oncogene Galpha13 mediates lysophosphatidic acid-mediated migration of pancreatic cancer cells. Pancreas 42, 819 (2013). 23508014 10.1097/MPA.0b013e318279c577 PMC3686977

[b19] Zhang J. X. . GNA13 as a prognostic factor and mediator of gastric cancer progression. Oncotarget 7, 4414 (2016). 26735177 10.18632/oncotarget.6780 PMC4826215

[b20] Grzelinski M. . Critical Role of G 12 and G 13 for Human Small Cell Lung Cancer Cell Proliferation *In vitro* and Tumor Growth *In vivo*. Clin Cancer Res 16, 1402 (2010). 20160064 10.1158/1078-0432.CCR-09-1873

[b21] Lappano R. & Maggiolini M. GPCRs and cancer. Acta Pharmacol Sin 33, 351 (2012). 22266725 10.1038/aps.2011.183 PMC4077134

[b22] Malchinkhuu E. . S1P(2) receptors mediate inhibition of glioma cell migration through Rho signaling pathways independent of PTEN. Biochem Biophys Res Commun 366, 963 (2008). 18088600 10.1016/j.bbrc.2007.12.054

[b23] Whitehurst B. . Anti-VEGF-A therapy reduces lymphatic vessel density and expression of VEGFR-3 in an orthotopic breast tumor model. Int J Cancer 121, 2181 (2007). 17597103 10.1002/ijc.22937

[b24] Tan W., Martin D. & Gutkind J. S. The Galpha13-Rho signaling axis is required for SDF-1-induced migration through CXCR4. J Biol Chem 281, 39542 (2006). 17056591 10.1074/jbc.M609062200

[b25] Bian D. . The G12/13-RhoA signaling pathway contributes to efficient lysophosphatidic acid-stimulated cell migration. Oncogene 25, 2234 (2006). 16301993 10.1038/sj.onc.1209261

[b26] Marinissen M. J., Servitja J. M., Offermanns S., Simon M. I. & Gutkind J. S. Thrombin protease-activated receptor-1 signals through Gq- and G13-initiated MAPK cascades regulating c-Jun expression to induce cell transformation. J Biol Chem 278, 46814 (2003). 12954641 10.1074/jbc.M305709200

[b27] Zhang J. X. . MiR-29c mediates epithelial-to-mesenchymal transition in human colorectal carcinoma metastasis via PTP4A and GNA13 regulation of beta-catenin signaling. Ann Oncol 25, 2196 (2014). 25193986 10.1093/annonc/mdu439

[b28] Hu Y., Xing J., Chen L., Zheng Y. & Zhou Z. RGS22 inhibits pancreatic adenocarcinoma cell migration through the G12/13 alpha subunit/F-actin pathway. Oncol Rep 34, 2507 (2015). 26323264 10.3892/or.2015.4209

[b29] Ha J. H. . Determinant role for the gep oncogenes, Galpha12/13, in ovarian cancer cell proliferation and xenograft tumor growth. Genes Cancer 6, 356 (2015). 26413218 10.18632/genesandcancer.72 PMC4575922

[b30] Radhika V., Onesime D., Ha J. H. & Dhanasekaran N. G 13 Stimulates Cell Migration through Cortactin-interacting Protein Hax-1. J Biol Chem 279, 49406 (2004). 15339924 10.1074/jbc.M408836200

[b31] Rutnam Z. J. & Yang B. B. The involvement of microRNAs in malignant transformation. Histol Histopathol 27, 1263 (2012). 22936445 10.14670/HH-27.1263

[b32] Zhang Z. J. & Ma S. L. miRNAs in breast cancer tumorigenesis (Review). Oncol Rep 27, 903 (2012). 22200848 10.3892/or.2011.1611 PMC3583555

[b33] Chen Z. . Activation of p115-RhoGEF requires direct association of Galpha13 and the Dbl homology domain. J Biol Chem 287, 25490 (2012). 22661716 10.1074/jbc.M111.333716 PMC3408165

[b34] Kozasa T., Hajicek N., Chow C. R. & Suzuki N. Signalling mechanisms of RhoGTPase regulation by the heterotrimeric G proteins G12 and G13. J Biochem 150, 357 (2011). 21873336 10.1093/jb/mvr105 PMC3218643

[b35] Muppidi J. R. . Loss of signalling via G α 13 in germinal centre B-cell-derived lymphoma. Nature 516, 254 (2014). 25274307 10.1038/nature13765 PMC4267955

