# Supplementary material for: High expression of GNA13 is associated with poor prognosis in hepatocellular carcinoma
Source: Sci Rep. 2016 Nov 24;6:35948. doi: 10.1038/srep35948 (PMC5121652; doi:10.1038/srep35948)

High expression of GNA13 is associated with poor prognosis in hepatocellular carcinoma

Yi Xu[1]*, Jian Rong[2]*, Shiyu Duan[3,4]*, Cui Chen[1], Yin Li[5], Baogang Peng[6], Bin Yi[2], Zhousan Zheng[1], Ying Gao[1], Kebing Wang[7], Miao Yun[8,9], Huiwen Weng[1], Jiaxing Zhang[1,8] & Sheng Ye[1]

[1]Department of Oncology, The First Affiliated Hospital, Sun Yat-Sen University, Guangzhou 510080, China, [2]Department of Extracorporeal Circulation, The First Affiliated Hospital, Sun Yat-sen University, Guangzhou 510080, China, [3]Department of Pathology, Nanfang Hospital, Southern Medical University, Guangzhou 510515, China, [4]Department of Pathology, School of Basic Medical Sciences, Southern Medical University, Guangzhou 510515, China, [5]Department of Gastrointestinal Surgery, The First Affiliated Hospital, Sun Yat-sen University, Guangzhou 510080, China, [6]Department of Liver Surgery, The First Affiliated Hospital, Sun Yat-sen University, Guangzhou 510080, China, [7]Department of Surgical Laboratory, The First Affiliated Hospital, Sun Yat-Sen University, Guangzhou 510080, China, [8]Sun Yat-sen University Cancer Center, State Key Laboratory of Oncology in South China, Collaborative Innovation Center for Cancer Medicine, Guangzhou 510060, China, [9]Department of Ultrasound, Cancer Center, Sun Yat-Sen University, Guangzhou 510060, China

Correspondence and requests for materials should be addressed to J.X.Z. (zjxlundy@hotmail.com) or S.Y. (yes20111212@163.com)

*These authors contributed equally to this work.

Supplementary figure legends

Supplementary Figure S1. Effect of GNA13 overexpression on the expression of P-AKT and P-ERK.

Western blotting analysis revealed that overexpression of GNA13 had no obvious effect on the protein levels of P-AKT and P-ERK.

Supplementary Figure S1

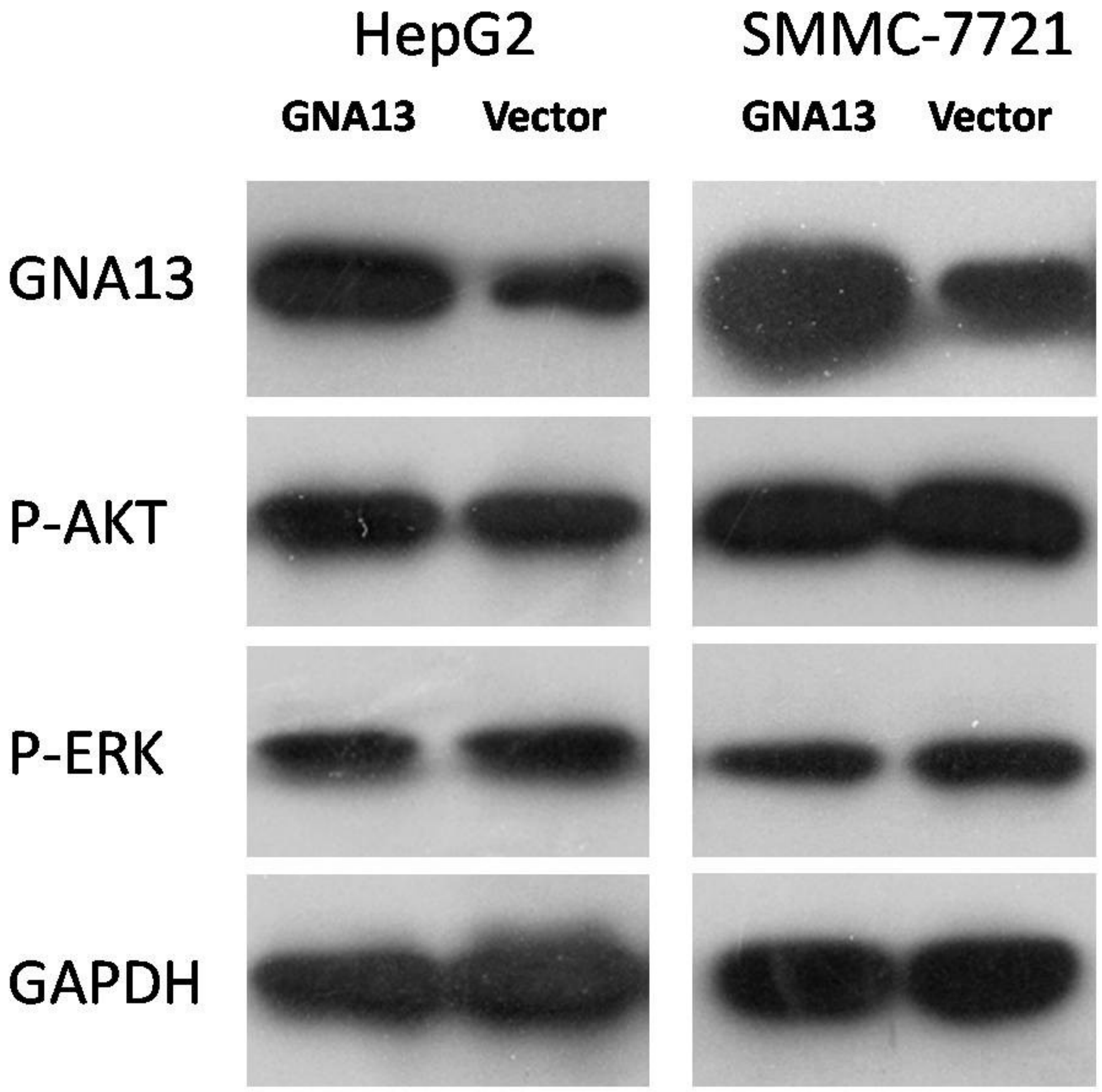

Supplement: Supplementary Information [file srep35948-s1.pdf]
